# ECHO-MPS, a dual modality strategy of cardiac imaging to identify myocardial ischemia

**DOI:** 10.1007/s10554-024-03257-x

**Published:** 2024-10-12

**Authors:** Cezary A. Szmigielski, Nikant Sabharwal, James D. Newton, Harald Becher

**Affiliations:** 1 Department of Cardiovascular Medicine, University of Oxford, Oxford, UK; 2 Department of Internal Medicine Hypertension and Vascular Diseases, Medical University of Warsaw, UCK CSK, 1A Banacha Street, Warsaw, 02-097 Poland; 3 Department of Cardiology, John Radcliffe Hospital, Oxford, OX3 9DU UK; 4 Division of Cardiology, Mazankowski Alberta Heart Institute, University of Alberta, Edmonton, Canada

**Keywords:** Coronary artery disease, Diagnostic imaging, Echocardiography, Myocardial perfusion scintigraphy

## Abstract

**Purpose:**

We aimed to evaluate an approach with resting echocardiography (TTE) and stress myocardial perfusion scintigraphy (MPS) compared to standard MPS in patients with stable angina and normal left ventricle (LV). We hypothesized that normal LV on TTE may allow for the elimination of rest MPS without compromising accuracy and offering an efficient diagnostic pathway with reduced radiation exposure.

**Methods:**

In a prospective, non-randomized study TTE was performed prior to MPS in patients (pts) referred for assessment of coronary artery disease (CAD). In pts with normal LV assessment was performed using the hybrid and the standard approach. TTE and MPS were interpreted by two TTE readers (ER1-2) and two MPS readers (NR1-2). ECHO-MPS was compared with standard MPS for diagnostic accuracy.

**Results:**

103 patients, mean age 61 *±* 12 year, (63 M, 40 W) were recruited. Standard MPS were normal in 75 patients and abnormal in 28 patients, with the hybrid approach 79 studies were reported as normal and 24 studies as abnormal. Kappa values were 0.580, (*p* < 0.001) for large, 0.394, (*p* < 0.001) for medium, and 0.298 (*p* = 0.002) for small defects. With standard MPS as a reference, sensitivity for detection of perfusion defects by ECHO-MPS was 75% (95% CI 0.67–0.83) [NR2] and 78% (95% CI 0.70–0.86)[NR1]. Specificity was 95% (95% CI 0.90–0.99) [NR2] and 95% (CI 95%CI 0.90–0.99) [NR1].

**Conclusions:**

ECHO-MPS protocol provides similar diagnostic accuracy as standard stress-rest MPS. In patients with normal systolic LV function in TTE, performing only stress MPS provides similar information as standard rest and stress MPS.

## Introduction

Stable angina in patients with coronary artery disease (CAD) is a common clinical presentation where accurate assessment of myocardial perfusion and left ventricular function is crucial for effective clinical management. Myocardial perfusion scintigraphy (MPS) using single photon emission computed tomography (SPECT) is a widely used method in the diagnosis and management of CAD, which has a confirmed prognostic value [1]. Traditionally, it involves the intravenous injection of a radionuclide tracer to evaluate myocardial perfusion at peak stress and a second dose after resting, normally separated by 3 to 48 h [2]. However, standard MPS exposes the patient to ionizing radiation, resulting in a small risk associated with the procedure, is resource-intensive and time-consuming for the patients and the medical providers [3]. The comparison of rest and stress images during MPS is necessary to differentiate fixed and reversible myocardial perfusion defects. A perfusion defect during stress but not at rest indicates myocardial ischemia. Perfusion defects at rest and during stress suggest a scar and are associated with reduced segmental myocardial function at rest. However, previous studies showed that regulation of coronary blood flow is a complex dynamic phenomenon and coronary flow reserve could be influenced by variations in both maximal vasodilation and resting coronary flow values [4–6]. In absence of obstructive coronary artery disease, risk factors can determine endothelial dysfunction able to influence both coronary vasodilation and resting coronary tone. However, in certain clinical scenarios, myocardial perfusion can be presumed to be normal. Specifically, in patients with stable angina who demonstrate normal global and regional wall motion at rest, the likelihood of significant coronary artery disease is often low [7]. Understanding these circumstances can aid in optimizing patient management by reducing unnecessary rest MPS diagnostic procedures and focusing on optimal clinical care. LV function can be accurately assessed with 2D/3D echocardiography in patients with adequate image quality. TTE with normal left ventricular global and regional function at rest may equate to normal perfusion at rest, given that this is the premise for stress echocardiography [7–9]. For this study, we hypothesized that in patients with stable angina and normal LV function, normal wall motion on TTE at rest could serve as a surrogate marker for normal myocardial perfusion at rest. This would allow for a diagnostic approach where resting TTE combined with stress MPS (ECHO-MPS) is as accurate as standard (stress and rest) MPS. The objective of our study was to compare the diagnostic accuracy of this hybrid ECHO-MPS approach with standard MPS, with the aim of streamlining the diagnostic process, improving patient care and optimizing the use of medical resources.

## Materials and methods

**Fig. 1:**
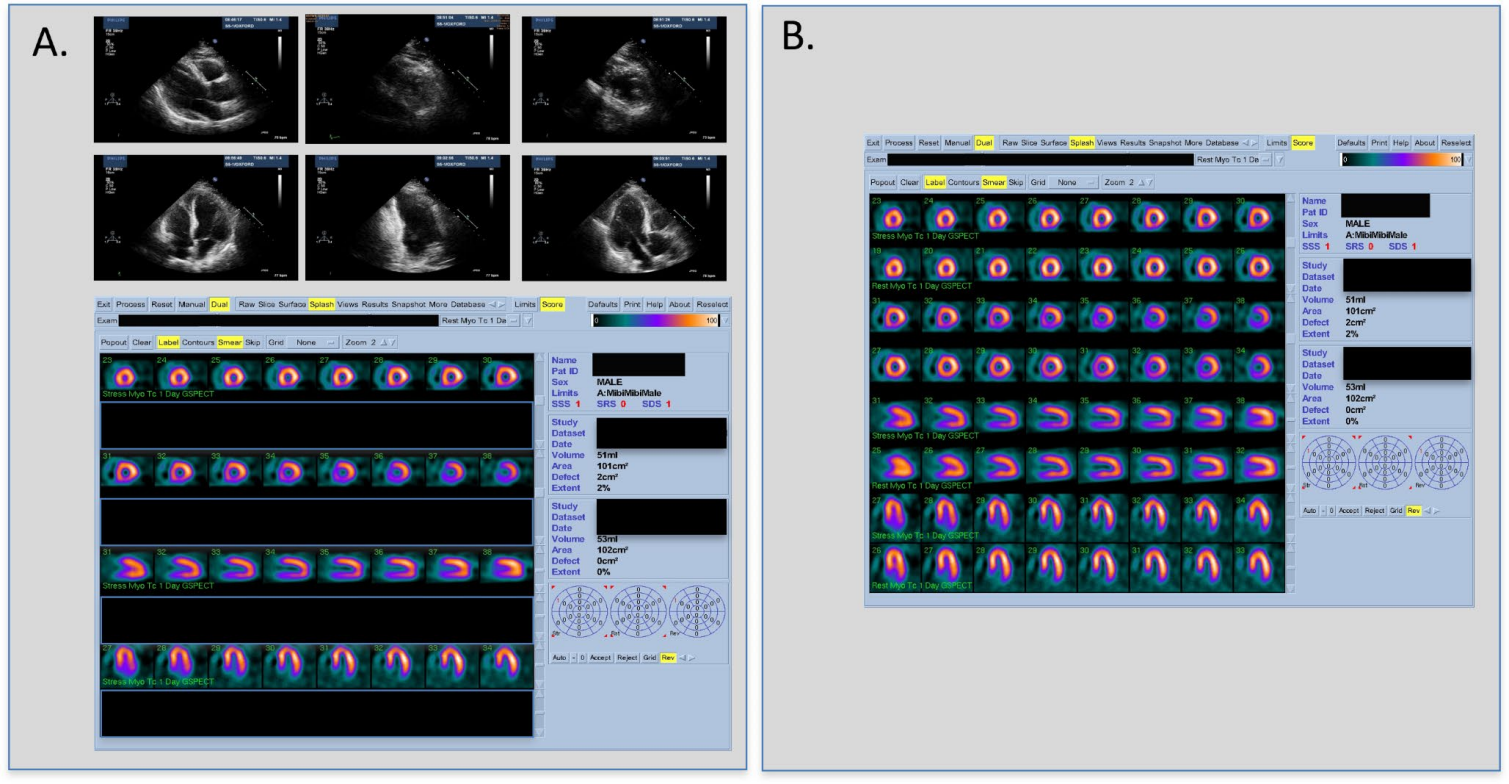
A dual modality cardiac strategy with echocardiography-nuclear assessment (ECHO-MPS) imaging and routine MPS in a patient. (Panel **A** - example of ECHO-MPS set of TTE images and stress SPECT images. Panel **B** – routine MPS with stress and rest SPECT images. Abbreviations: MPS, myocardial perfusion scintigraphy; SPECT single photon emission computed tomography; TTE, transthoracic echocardiogram

This was a prospective, non-randomized study comparing a new imaging protocol (rest echo-stress nuclear, ECHO-MPS) to conventional MPS (stress and rest SPECT) (Fig. 1). The primary objective of this study was to evaluate the ability of ECHO-MPS to assess myocardial perfusion in comparison with conventional MPS. The secondary objective was to evaluate the ability of an ECHO-MPS study to identify the territory of myocardial ischemia when compared to standard MPS. Participants were identified from consecutive referrals for MPS at the John Radcliffe Hospital, Oxford, United Kingdom. The patients were enrolled in this study if they were referred for a standard MPS, male or female; aged 18 years or above. The participants were not eligible to enter the study if they had acute coronary syndrome (ACS), inadequate TTE image quality or a contraindication to MPS (significant arrhythmia, e.g., ventricular tachycardia, second-or third-degree atrioventricular block, sinus bradycardia less than 45 beats per minute, systolic blood pressure less than 90 mm Hg or above 200 mm Hg, diastolic blood pressure above 110 mm Hg, broncho-constrictive disease, known hypersensitivity to vasodilator agent). The study was reviewed and approved by the local Ethics Committee and patients gave informed consent for echocardiographic examination.

### Echocardiography

A comprehensive 2D transthoracic echocardiogram (TTE) was performed in the standard manner using a commercially available ultrasound scanner (Philips iE 33, Philips Ultrasound, Bothell, USA), with images transferred to an off-line digital workstation with Xcelera software (Philips Healthcare, Koninklijke Philips Electronics N.V., The Netherlands) for storage and reporting. DICOM files were recorded and stored on DVD. The recordings were performed by an experienced sonographer according to the guidelines [10, 11]. The patients were scanned on the day of the stress SPECT study, therefore there was no interval between TTE and SPECT to reduce potential impact of the interval on the results. Participants needed to have well visualized myocardium in at least 2 apical windows for reliable regional wall motion assessment. Standard echocardiographic images were obtained in the following views: parasternal long axis (PLAX), parasternal short axis (PSAX), apical 4-chamber (A4C), apical 3-chamber (A3C) and apical 2-chamber (A2C). Analysis was based on the 17–segment model recommended by the American Society of Echocardiography (ASE) [11]. Visual assessment of the image quality and regional wall motion analysis was evaluated using the standard grading scale by two echo readers (ER1 and ER2).

### Myocardial perfusion scintigraphy (MPS)

A one day stress/rest MPS protocol was implemented on the gamma camera Pulse CDC Compact (IS2 Medical Systems, Ontario, Canada), with 1000 MBq (technetium-99 m, Sestamibi) in two injections (300 + 700 MBq) on a collimator according to standard guidelines [12]. Left ventricular (LV) ejection fraction (EF) and volumes, as well as reginal wall motion and thickening were computed routinely using commercially available software (Quantitative Perfusion SPECT, QPS, Cedars-Sinai Medical Center, Los Angeles, USA).

The SPECT images were visually interpreted in all 3 standard projections, along with the gated SPECT and raw image data were assessed for quality and to determine the size of the defect and whether defects were fixed, reversible, or mixed. Defect extent was categorized according to the percentage of the overall myocardium involved. The size of defects was defined as: large (> 20% of myocardium, equivalent to *≥* 4 segments), medium (15–20% of myocardium, 2–3 segments) and small defects (5–10% of myocardium, 1–2 segments). Presence of artefacts was assessed. The agreement in the judgement of regional perfusion patterns between nuclear reader 1 and 2 (NR1 and NR2) was evaluated. Nuclear Reader 2 was evaluating the scans as a routine clinical read in the nuclear medicine laboratory.

### Statistical analysis

Continuous variables are expressed as mean *±* SD, and categorical variables are expressed as frequency. Sensitivity and specificity were derived according to standard definitions and are presented with the corresponding 95% confidence intervals (CI). The values were calculated to assess the diagnostic performance of the new imaging approach (ECHO-MPS) in comparison to the standard MPS as the reference. Sensitivity measured the proportion of patients with CAD who were correctly identified by the new technique as having the disease. This was calculated by dividing the number of true positive cases (patients identified as having CAD by both the new technique and the reference) by the total number of patients who had CAD, including both true positives and false negatives. Specificity reflected the proportion of patients without CAD who were correctly identified as disease-free by the new technique. This was determined by dividing the number of true negative cases (patients identified as not having CAD by both the new technique and the reference) by the total number of patients who did not have CAD, including both true negatives and false positives. Comparison between standard MPS and ECHO-MPS between reports was performed using kappa statistics. Statistical analyses were performed with SPSS (version 15.0, IBM SPSS Statistics, IBM Corporation, USA). A p-value of < 0.05 was considered significant.

## Results

We studied 103 patients: mean age 61 *±* 12 year, 63 men (61%), 40 women (39%). The majority were referred for diagnosis of stable coronary artery disease (90 pts., 87%), other reasons included: evaluation of known CAD (7 pts., 7%), assessment of acute chest pain (3 pts., 3%), assessment before solid organ transplant (3 pts., 3%). Echocardiographic image quality was identified as good in 53 cases (51%), intermediate in 33 cases (32%) and poor in 17 cases (17%). Left ventricular ejection fraction was normal (above 60%) in both imaging modalities. ECHO-MPS results were normal in 79 pts (77%), and abnormal in 24 pts (23%). MPS results were normal in 75 pts (73%), abnormal in 28 pts (27%) according to reader NR1, and normal in 73 pts (71%), and abnormal in 30 pts (29%), according to reader NR2, with Kappa 0.650, *p* < 0.01, and Kappa 0.650, *p* < 0.01, for normal and abnormal perfusion, respectively (Table 1). With ECHO-MPS approach, 29 defects were identified, with MPS (NR1) 32 defects: reversible 18 (56%), fixed 6 (19%), mixed 8 (25%) and 36 defects with MPS (NR2), reversible 18 (50%), fixed 4 (11%), mixed 14 (39%). ECHO-MPS identified 24 patients with defects, including 6 pts. (25%) with large defects, 7 pts. (29%) with medium defects and 11 pts. (46%) with small defects. MPS NR1 identified 28 pts with defects, among them 6 pts. (21%) with large defects, 9 pts. (32%) with medium defects and 13 pts. (47%) with small defects. MPS NR2 showed 30 patients with defects, including 4 pts. (13%) with large defects, 11 pts. (37%) with medium defects and 15 pts. (50%) with small defects. Kappa values were 0.580, (*p* < 0.001) for large defects, 0.394, (*p* < 0.001) for medium defects, and 0.298 (*p* = 0.002) for small defects (Table 2). Transthoracic echocardiography (TTE) identified regional wall motion abnormalities (RWMA) in 11 pts. (11%) by ER1 and in 10 pts. (10%) by ER2, with Kappa 0.735, *p* < 0.001. Normal wall motion was assessed in 92 pts (89%) by ER1 and in 93 pts (90%) by ER2, with Kappa 0.735, *p* < 0.001 (Table 3). Overall sensitivity and specificity were calculated for CAD diagnosis by standard MPS as a reference test for comparison. With this approach, sensitivity for ECHO-MPS was 75% (95% CI 0.67–0.83) vs. NR2 and 78% (95% CI 0.70–0.86) vs. NR1. Specificity was 95% (95% CI 0.90–0.99) for NR2 and also 95% (CI 95%CI 0.90–0.99) for NR1. The ECHO-MPS was able to identify all large defects. During follow-up of 12.9 years, we found records of 16 deaths in the studied group, therefore the mortality was around 15% in our population. There were differences between the ECHO-MPS and nuclear readers in 13 patients. Artifacts were noted in MPS studies in all groups (NR1 18 patients, NR2 45 patients, ECHO-MPS 27 patients). In 8 patients ECHO-MPS was interpreted as normal or with artefacts but one of the nuclear readers described small or very small abnormalities. In one patient ECHO-MPS was normal with diaphragm artefact and in a standard nuclear (NR2) report perfusion was normal with inferior soft tissue attenuation, and in a nuclear read (NR1) there was normal perfusion and inferior hypokinesis on GSPECT. In 4 patients ECHO-MPS was reported as abnormal, but one of the nuclear readers described abnormalities and another nuclear reader reported normal result.

**Table 1:** Comparison of perfusion assessment with ECHO-MPS and standard MPS

Study	ECHO-MPS [*n*, (%)]	MPS NR1 [*n*, (%)]	MPS NR 2 [*n*, (%)]	Kappa	*P* value
Perfusion normal	79 (77)	75 (73)	73 (71)	0.650	< 0.01
Perfusion abnormal	24 (23)	28 (27)	30 (29)	0.650	< 0.01
Number of defects	29	32	36		
Defects reversible	N/A	18 (56)	18 (50)		
Defects fixed	N/A	6 (19)	4 (11)		
Defects mixed	N/A	8 (25)	14 (39)		

Abbreviations: MPS, myocardial perfusion scintigraphy; n, number of patients; N/A, non-applicable; NR, nuclear reader

**Table 2:** Number of patients with different defects sizes assessed with ECHO-MPS and MPS

Defects, myocardial involvement	ECHO-MPS [*n*, (%)]	MPS NR1 [*n*, (%)]	MPS NR2 [*n*, (%)]	Kappa	*P* value
Small (5–10%) (= 1–2 segments)	11 (46)	13 (47)	15 (50)	0.298	0.002
Medium (15–20%) (= 2–3 segments)	7 (29)	9 (32)	11 (37)	0.394	< 0.001
Large (> 20%) (*≥* 4 segments)	6 (25)	6 (21)	4 (13)	0.580	< 0.001
Patients with defects, all [n]	24	28	30	N/A	N/A

Abbreviations: MPS, myocardial perfusion scintigraphy; n, number of patients; N/A, non-applicable; NR, nuclear reader

**Table 3:** Comparison of wall motion assessment with resting echocardiography

Study	TTE ER 1 [*n*, (%)]	TTE ER 2 [*n*, (%)]	Kappa	*P* value
Normal wall motion	92 (89)	93 (90)	0.735	< 0.001
RWMA	11 (11)	10 (10)	0.735	< 0.001
Number of segments				
RWMA segments	25	28		
Hypokinetic segments	15 (60)	17 (61)		
Akinetic segments	10 (40)	11 (39)		
Dyskinetic segments	0	0		

Abbreviations: ER, echo reader; n, number of patients; N/A, non-applicable; RWMA, regional wall motion abnormalities

## Discussion

**Fig. 2:**
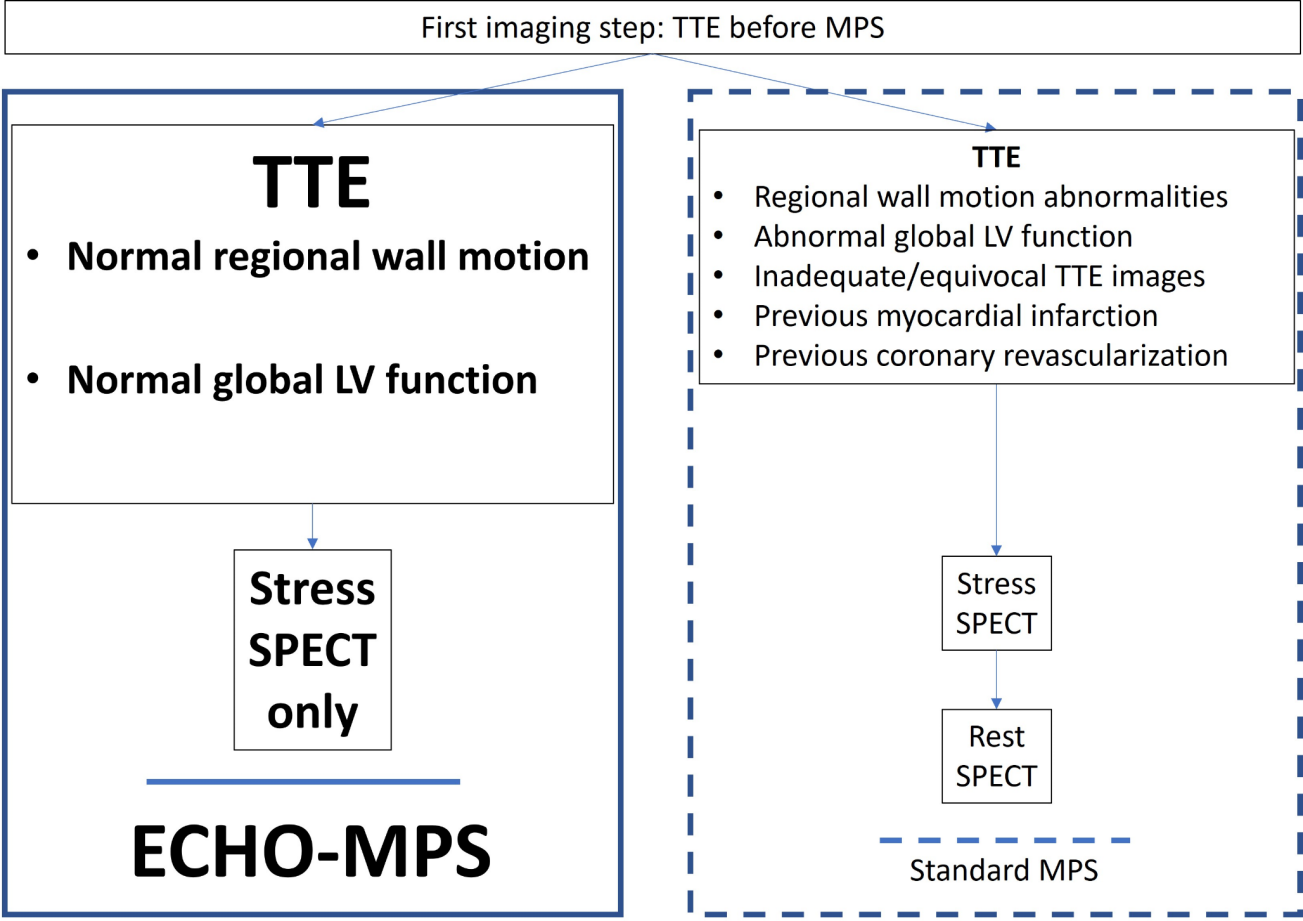
Proposal for a hybrid echocardiography-nuclear assessment (ECHO-MPS) to risk stratify patients with CAD. Abbreviations: MPS, myocardial perfusion scintigraphy; SPECT single photon emission computed tomography; TTE, transthoracic echocardiogram

In this study we demonstrated that a dual modality imaging including a baseline rest transthoracic echocardiogram (TTE) combined with a stress MPS (the ECHO-MPS protocol) showed similar diagnostic accuracy as an established stress-rest MPS (Fig. 2). The ECHO-MPS allowed integration of complementary information available from two established non-invasive imaging modalities used for diagnosis and follow-up of CAD [13, 14]. We compared assessment from ECHO-MPS with similar number and localization of defects in MPS. Notably, the ECHO-MPS showed especially good diagnostic accuracy for large and medium defects, which are clinically important, and we showed that they can be adequately assessed with this new protocol. Therefore, the ECHO-MPS approach allows similarly good prognostication of patients evaluated for CAD to standard stress-rest MPS, which has a confirmed incremental prognostic value [15]. Large pool of data showed before, that when MPS is normal, the prognosis is good, and that overall mortality from adverse cardiac events is low [15]. MPS continues to be recognized as an important modality in the management of patients with chest pain. It has Level 1 Category of Recommendation (LOR) for intermediate-risk patients with acute or chronic chest pain and no known CAD even in the newest AHA/ACC chest pain guidelines [16]. In our small study, but with a median follow-up of 12.9 years, we showed that mortality was in the range of 15%, with 16 deaths recorded in the studied group. Our study’s long-term mortality data in patients with intermediate risk of coronary artery disease align with broader findings from the literature. Some large studies showed mortality rates that could be extrapolated for similar time of follow-up. In the Framingham Heart study, mortality data stratified by age and risk factors, showing that individuals in the 60–69 age group with intermediate risk had a 10-year mortality rate around 15–18% [17]. Large international registry REACH showed high 1-year event rates that accrued almost linearly over time. For patients aged 55–64 with intermediate risk, the mortality rate over a 4-year period was approximately 6–7%. Projecting this over a longer period, similar mortality rates (around 15–20% over 10–12 years) could be expected [18]. However, in their study Duvall et al. showed a low annualized cardiac event rate (< 1%) among 10,609 patients with a normal MPS [19]. Large trials such as the ISCHEMIA trial, demonstrated no substantial difference in outcomes between initial invasive or conservative strategies even in patients with severe ischemia [20, 21]. However, our study did not collect specific therapeutic strategies employed, or detailed patient outcomes. Therefore, we cannot fully explore the impact of these factors on patient prognosis. For our study, we included only patients with no more than intermediate cardiovascular risk, because these patients would be expected to have higher rate of normal rest TTE, compared to high-risk patients. Our study was designed and started before coronary computed tomography angiography (CCTA) was available for a study to include CAD anatomic assessment. However, in the meantime, the role of anatomic imaging techniques has been growing, especially CCTA. But, given their often limited availability, and substantial cost, there is still need for large volume cardiovascular imaging techniques like TTE and MPS. Additionally, our study was performed without accessibility to positron emission technology (PET) technology. PET studies have demonstrated that regulation of coronary blood flow is a complex dynamic phenomenon and coronary flow reserve could be influenced by variations in both maximal vasodilation and resting coronary flow values. In absence of obstructive coronary artery disease, risk factors can determine endothelial dysfunction able to influence both coronary vasodilation and resting coronary tone. In example, myocardial hypertrophy, often associated to hypertension, can determine low resting perfusion values despite normal wall motion [4]. In presence of CAD, PET studies demonstrated resting coronary flow values lower than in normal subjects in regions supplied by not-stenotic coronary arteries and normal regional wall motion [5]. Moreover, some patients may have normal wall motion after myocardial infarction [6]. Our data provide evidence of the safety and efficacy of MPS stress only imaging in patients with normal TTE and provide further reassurance of the MPS rest-image omission. However, we did not assess the MPS stress-only approach. To our knowledge, all studies evaluating a stress-only imaging protocol used some form of attenuation correction (AC). A successful utilization of stress-only imaging requires the application of AC capabilities including hardware and software or CT and post-processing iterative reconstruction that was not available for this study [22, 23]. AC can be especially helpful to discern mild abnormalities suspicious for diaphragmatic or breast attenuation [24]. Without AC attenuation artefacts are commonly observed on stress-only studies with a prevalence of 50–78% [25]. However, increased cost of additional hardware and software for AC preclude it from being available in some centers. In our study we used commercially available post-processing MPS software. Further development in sophisticated software reconstruction methods can improve image quality obtained by standard SPECT cameras, which can be exploited to reduce imaging time or radiation dose without replacement of the system with a new costly scanner [26]. Similarly, newer CZT (Cadmium-Zinc-Telluride) gamma cameras offer several advantages, including enhanced image quality, lower radiation doses, and faster acquisition times. However, their high cost, limited availability, need for specialized training, and technological limitations pose significant challenges to their widespread adoption. When considering the implementation of any of mentioned new technologies, these limitations must be weighed against the potential clinical benefits to determine the best approach for patient care. On the other hand, stress echocardiography is a valuable tool in the non-invasive assessment of coronary artery disease and myocardial function, offering several advantages such as no radiation exposure and real-time imaging. However, stress echocardiography is much more demanding from the operator and interpreter point of view than rest echocardiography. Therefore, its limitations, such as operator dependency, technical challenges, limited acoustic windows, and diagnostic variability, especially without contrast, must be considered when choosing the most appropriate imaging modality for a particular patient. However, it is important to note that the use of advanced instrumentation such as CZT gamma cameras or non-ionizing protocols like stress echocardiography can offer even greater levels of safety by further reducing or eliminating radiation exposure. The selection of the most appropriate imaging modality should consider these factors in conjunction with the specific clinical scenario. We believe that, combining rest echocardiography with stress MPS may help mitigate these limitations. A simple bi-modality approach with ECHO-MPS protocol can help imaging centers with TTE and MPS, but no access to AC or CZT or expert stress echocardiography. Standard stress and rest MPS images are still an important practice in high volume cardiology centers. Some authors suggest that routine stress-rest MPS protocols should be reserved for patients with known CAD and prior MI or LV dysfunction. In this setting it is easier to differentiate reversible from fixed defects, recognize artefacts, transient LV dilatation and stunning. It is also more accurate for left ventricular ejection fraction measurement in cases of imperfect gating. Also, resting MPS can be useful in cases with normal TTE and abnormal stress MPS. MPS can sometimes detect perfusion abnormalities in the setting of microvascular disease, while TTE typically does not detect microvascular disease because it does not cause gross structural changes or significant resting wall motion abnormalities. Resting MPS can be important in an uncommon clinical scenario when a patient having had a MI yet showing normal wall motion on TTE in the affected region. This may potentially occur when the ischemic area is small, subendocardial, rapidly reperfused or if collateral circulation is sufficient to preserve myocardial function despite local occlusion. However, the ECHO-MPS can still provide the opportunity to decrease the amount of radiation used in a routine stress-rest MPS, while preserving the diagnostic power. It can also reduce tracer usage, overall costs, time, improve patient convenience and can potentially increase imaging laboratory volume and efficiency. The necessity of TTE before performing MPS may be seen as limiting factor in settings where access to echocardiography equipment may be limited. However, with the advent of point-of-care ultrasound (POCUS) and the increasing availability of compact, handheld ultrasound devices, the integration of echocardiography into routine practice is becoming more feasible. As these handheld ultrasound devices become more prevalent, future studies could focus on validating the use of POCUS in combination with MPS in diverse clinical settings. This would not only broaden the applicability of our findings but also help establish POCUS as a potential part of cardiac assessment prior to MPS. Therefore, our study can help in further developing imaging strategies. It can also help adapting cardiac imaging to fast evolving handheld technology. Whatever the echocardiographic hardware may be, the ideal candidates for ECHO-MPS imaging are patients with no known coronary artery disease and if CAD is present, without prior history of a myocardial infarction or coronary revascularization procedure [27]. In our study, there were differences between the ECHO-MPS and nuclear readers in 13 patients. Artifacts were noted in MPS studies in all groups. The differences observed between the ECHO-MPS, and nuclear readers appear to be largely due to the presence of artifacts and the subjective interpretation of small and subtle abnormalities. The discrepancies may stem from differences in the sensitivity of the modalities and/or the subjective thresholds of the readers for detecting and reporting the smallest defects. These inconsistencies highlight the challenge of interpreting imaging results in the presence of artifacts, which can mimic or obscure true abnormalities. The differences could therefore be attributed to the varying degrees of impact that these artifacts have on the imaging modalities and the interpretive differences among readers. Despite the challenges, the findings of our study, which demonstrated that a hybrid diagnostic approach using resting TTE and stress MPS (ECHO-MPS) offers comparable accuracy to standard MPS, can potentially be generalized to a broader cardiology patient population. Our study included patients with stable angina and normal LV function, which is representative of a significant subset of patients in general cardiology practice. Potentially, similar diagnostic accuracy could be achieved in a more diverse population. However, further studies involving a broader patient demographic, including those with reduced LV function or other coexisting cardiovascular conditions, would be necessary to fully validate these findings. If confirmed, the adoption of this streamlined approach could enhance diagnostic efficiency and reduce unnecessary imaging in a wider clinical context, thereby optimizing resource utilization and patient care across cardiology practices.

Based on the results of our study, we suggest that the ECHO-MPS protocol present here can be helpful in cardiovascular testing in large number of patients with CAD. Our study strengthens the concept that even simple cardiac imaging tests tend to be complementary. Careful patient selection, physician and technical local expertise, equipment availability, and patient preference are all important factors to consider for the concept of multimodality, patient first imaging [28].

### Limitations of the study

This is a study performed on a small population of 103 patients, which impacts the validation of ECHO-MPS. A larger cohort would strengthen the validation. However, we believe our study still provides valuable insights into this emerging diagnostic approach. Even with a small cohort we performed a prospective, controlled, single center study, with a mix of male and female patients in a range of ages typically referred for cardiac tests, which can offer clinically relevant information. We designed our study with a focused aim of comparing the results of SPECT and echocardiography imaging modalities. As such, we focused on the imaging modalities and did not collect detailed clinical data. Future studies can expand on our findings by incorporating detailed clinical data to further explore relationship between imaging results and patient characteristics. In our study, although we were able to track mortality over a median follow-up of 12.9 years, we did not plan for detailed survival analysis. As a result, we could not differentiate between cardiovascular and non-cardiovascular deaths, which limits our ability to draw specific conclusions about the relationship between baseline risk factors and mortality outcomes in our cohort. Despite these limitations, the mortality data we report provide some insights into long-term outcomes in a population with intermediate risk of coronary artery disease. We used a clinical standard MPS as a reference test for comparison. Because the number of patients who need interventional assessment was very low, there was no option to include invasive coronary angiography as obligatory verification of coronary disease, and it would be unethical to routinely perform invasive procedures at this stage of assessment. As a consequence of the absence of the coronary anatomy of the studied population, we obtained a comparison between the hybrid ECHO-MPS approach and MPS, but not the relative accuracy of the two protocols in our population. Coronary computed tomography angiography was not available during designing of the study. We did not use echocardiographic contrast, that improve myocardial delineation [29], strain imaging, nor three dimensional echocardiography [30, 31]. Automated artificial intelligence systems to augment imaging analysis and clinical interpretation, such as used recently for imaging stress testing were not existent at the time of the study [32].

## Conclusion

A hybrid study combining a rest echocardiogram with stress nuclear scan (ECHO-MPS) provides similar diagnostic accuracy as standard stress-rest MPS. Most stress perfusion scans can be read without comparison to rest perfusion, if adequate TTE is available. In patients with normal systolic LV function in TTE, performing only stress nuclear provides similar information as standard nuclear imaging with both stress and rest recordings.
